# Assessment of Early Cardiotoxicity and Cardiac Dysfunction of Radioligand Therapy in Patients with Neuroendocrine Tumors

**DOI:** 10.3390/cancers17193219

**Published:** 2025-10-02

**Authors:** Katarzyna Jóźwik-Plebanek, Marek Saracyn, Maciej Kołodziej, Weronika Mądra, Adam Daniel Durma, Mirosław Dziuk, Zuzanna Balcerska, Katarzyna Janiak, Katarzyna Gniadek-Olejniczak, Grzegorz Kamiński

**Affiliations:** 1 Department of Endocrinology and Radioisotope Therapy, Military Institute of Medicine—National Research Institute, 04-141 Warsaw, Poland; msaracyn@wim.mil.pl (M.S.); mkolodziej@wim.mil.pl (M.K.); wmadra@wim.mil.pl (W.M.); zbalcerska@wim.mil.pl (Z.B.); kjaniak@wim.mil.pl (K.J.); gkaminski@wim.mil.pl (G.K.); 2 Faculty of Medicine, Warsaw University, 00-927 Warsaw, Poland; 3 Nuclear Medicine Department, Military Institute of Medicine—National Research Institute, 04-141 Warsaw, Poland; mdziuk@wim.mil.pl; 4 Neurorehabilitation Clinic, Military Institute of Medicine—National Research Institute, 04-141 Warsaw, Poland; kgniadek-olejniczak@wim.mil.pl

**Keywords:** neuroendocrine neoplasms, neuroendocrine tumors, NEN, RLT, PRRT, 177-Lu, 90-Y, complications, adverse events, cardiotoxicity, peptide receptor radionuclide therapy

## Abstract

**Simple Summary:**

Radioligand therapy (RLT) with [^177^Lu]Lu-DOTA-TATE, alone or in combination with [^90^Y]Y-DOTA-TATE, is an established treatment option for patients with neuroendocrine tumors (NETs). Although highly effective, concerns remain regarding its potential impact on cardiac injury, particularly in subgroups with increased susceptibility to such injury. This study evaluated potential cardiotoxicity by measuring serum concentrations of troponin I, CK-MB, and NT-proBNP before and after RLT. A total of 60 patients undergoing 228 treatment courses were included, representing the largest cohort analyzed to date. No significant increases in cardiac biomarkers were observed, either in the overall population or in high-risk subgroups such as those with heart failure, carcinoid heart disease, or prior chemotherapy. These findings provide the first evidence from a large patient population that RLT is not associated with cardiotoxicity or cardiac dysfunction. Our results therefore support the cardiac safety of RLT in NET patients, including those at increased risk of cardiotoxicity.

**Abstract:**

**Background**: Cardiotoxicity remains a concern across cancer therapies. To date, there is a lack of extensive studies evaluating the potential impact of radioligand therapy (RLT) on myocardial injury in patients with neuroendocrine tumors (NETs), particularly in subgroups with increased susceptibility to such injury. This study aimed to assess the potential cardiotoxic effects and myocardial dysfunction associated with RLT using both [^177^Lu]Lu-DOTA-TATE and tandem therapy with [^177^Lu]Lu-DOTA-TATE/[^90^Y]Y-DOTA-TATE in patients with NETs, including specific high-risk subgroups such as patients with pre-existing heart failure, carcinoid heart disease or those previously treated with chemotherapy, by monitoring serum concentration of troponin I, CK-MB, and NT-proBNP before and after RLT. **Methods**: We conducted a retrospective observational analysis of 60 consecutive NET patients who underwent 228 RLT courses. A comprehensive cardiac assessment, including a detailed medical history, was performed. Additionally, serum troponin I, CKMB and NT-proBNP concentrations were measured prior to treatment and 48 h post-therapy. Fifty-two patients received [^177^Lu]Lu-DOTA-TATE monotherapy, while eight patients were treated with tandem therapy. **Results**: No increase in cardiotoxicity markers was observed in the overall study population following RLT administration (ΔTroponin −0.2 [−1.4–0.3]ng/L, *p* = 0.007; ∆CKMB 0.0 [−4.0–3.0]U/L, *p* = 0.90; ΔNT-proBNP 4.0 [−45.6–33.6]pg/mL) as well as in the subgroup receiving tandem therapy (ΔTroponin 0.7 [−1.7–013]ng/L, *p* = 0.68; ΔCKMB −0.5 [−10.7–3.0]U/L, *p* = 0.21; ΔNT-proBNP −21.6 [−44.1–16.7]pg/mL). Furthermore, none of the predefined patient subgroups exhibited signs of cardiotoxicity or evidence of myocardial dysfunction. **Conclusions**: RLT is a safe anticancer treatment option for patients with NETs in terms of cardiotoxicity and cardiac dysfunction, including those at higher risk of cardiovascular complications.

## 1. Introduction

Radioligand therapy (RLT), a form of targeted radionuclide therapy, is widely employed in the treatment of neuroendocrine tumors (NETs). Radiolabelled somatostatin analogs—most commonly conjugated with lutetium-177 (^177^Lu)—emit medium-energy beta particles, which induce DNA damage and subsequent apoptosis or necrosis of target cells. While RLT effectively targets somatostatin receptor-expressing NET cells, large, randomized trials have demonstrated that it may also cause direct off-target toxicity—particularly to the hematopoietic system and the kidneys, with reported complication rates of up to 10–20% [1,2,3]. However, to date, there is a lack of systematic data addressing the potential cardiotoxic effects of RLT.

For approximately a decade, national and international cardiology societies have been regularly publishing cardio-oncology guidelines addressing the principles of patient qualification, potential cardiotoxic effects, and recommendations for monitoring cardiovascular (CV) complications associated with various forms of cancer therapy [4,5,6]. These include conventional chemotherapies (CHT) (e.g., anthracyclines), targeted therapies (e.g., tyrosine kinase inhibitors (TKI)), immunotherapies, and external beam radiotherapy. However, RLT has not yet been included in any of the currently published cardio-oncology guidelines, primarily due to the limited number of studies assessing its CV effects. In clinical practice and in selected literature reviews, cardiac monitoring during RLT is recommended in NET patients, particularly those with carcinoid syndrome (CS) or pre-existing cardiac conditions; however, these are considered expert-based suggestions rather than formal guideline recommendations [7].

Among the various forms of cardiotoxicity defined in the 2022 International Cardio-Oncology Society (IC-OS) consensus statement, particular attention is given to the issue of cardiac dysfunction [8]. According to this definition, cardiac dysfunction can be classified into symptomatic heart failure (HF) and asymptomatic cardiac dysfunction. Besides clinical symptoms and assessment of left ventricular ejection fraction (LVEF) by echocardiography, more sensitive methods to detect and confirm cardiac dysfunction should be considered, including global longitudinal strain (GLS) and/or cardiac biomarkers (e.g., troponins and natriuretic peptides). Troponin elevation above the 99th percentile cut-off for the specific assay used can serve as a supportive biomarker indicating cardiac injury, while an increase in natriuretic peptide levels compared to baseline prior to therapy may confirm the onset of new or worsening pre-existing HF. Isolated elevations of these biomarkers without corresponding clinical and imaging changes may be considered biochemical evidence of cardiotoxicity [8].

The potential cardiotoxic effects of RLT in NET patients may include both direct and indirect mechanisms. Direct injury can result from radiation-induced damage to myocardial and vascular cells. Indirect effects may involve fluid overload of the circulatory system during treatment, as more than 3000 mL of intravenous fluids are typically administered over a 2-day period during RLT for NET. Additional potential negative effects may include arrhythmias and blood pressure dysregulation related to the administration of somatostatin analogs conjugated with radionuclides, as well as hemodynamic instability caused by the release of bioactive substances (e.g., hormones such as serotonin, bradykinins, and cytokines) from injured NET cells.

The potential of ionizing radiation to adversely affect the CV system has been unequivocally demonstrated [9,10]; however, there is still uncertainty regarding the effects of ionizing radiation exposure resulting from radiopharmaceutical therapies.

In patients with thyroid cancer treated with radioiodine—due to the large patient population, generally favorable prognosis, and the frequent absence of concomitant cardiotoxic therapies—it has been potentially easier to evaluate the short- and long-term CV consequences of radioisotope therapy. Unfortunately, early cardiotoxicity of radioiodine therapy in patients with thyroid cancer has not been systematically evaluated to date. Although the results of long-term observational studies also remain inconclusive, there is growing evidence to support the association between radioiodine treatment and an increased incidence of long-term CV complications [11,12,13,14]. As suggested by Kao et al., both CV morbidity and mortality among patients treated with radioiodine appear to be associated with the cumulative administered activity [13].

It is particularly important to emphasize that, in contrast to radioiodine therapy for thyroid cancer—where, in most patients, the primary accumulation of radioactivity occurs in the remnants of thyroid tissue within the postoperative bed—RLT in NETs typically involves disseminated disease. As a result, there is a significantly higher number of radiotracer uptake sites throughout the body, which may lead to increased exposure of non-target tissues to the potentially damaging effects of ionizing radiation.

According to dosimetric data, the estimated absorbed dose to the cardiac wall during RLT with [^177^Lu]Lu-DOTA-TATE is comparable to that received by the bone marrow [15]. This observation—especially considering the well-documented myelotoxic effects of RLT—raises a legitimate concern regarding the potential cardiotoxicity of this form of “internal radiotherapy”.

In the context of RLT, available data regarding cardiotoxicity and the impact of treatment on CV complications remain exceptionally limited. Only isolated cases of cardiotoxic effects have been reported to date. For instance, Hendifar et al. described a case of persistent cardiomyopathy following just two courses of [^177^Lu]Lu-DOTA-TATE therapy [16]. In the currently available literature, the direct effect of RLT on myocardial injury in patients with NETs, assessed by measuring cardiac troponin I concentration—a sensitive biomarker of myocardial injury—has been evaluated in only one study conducted on a very small cohort of 13 patients, which did not demonstrate an increase in troponin I levels following treatment [17]. A definitive assessment of the potential cardiotoxicity in specific patient subgroups, such as those with coexisting HF, prior CHT, or diagnosed carcinoid heart disease (CHD), has never been performed.

When considering the cardiotoxicity of applied treatments, it is important to remember that patients with NETs often receive multiple lines of therapy besides RLT, including CHT, TKI, mammalian Target of Rapamycin inhibitors (mTOR), and potentially other novel therapies such as immune checkpoint inhibitors (ICIs) at various stages of treatment. Given the known cardiotoxic potential of some of these therapies—such as sunitinib, everolimus, or CHT regimens based on capecitabine/temozolomide (CAPTEM)—understanding the safety profile of RLT regarding cardiotoxicity may prove crucial in therapeutic decision-making [18,19,20,21].

This study aimed to collect data on the potential early cardiotoxicity of RLT using [^177^Lu]- and [^90^Y]-labeled somatostatin analogs in patients with NETs, based on sensitive biomarkers of cardiomyocyte injury such as troponin I and, additionally, the Creatine Kinase–MB isoenzyme (CK-MB). Additionally, the study evaluated the potential for cardiac dysfunction secondary to hemodynamic overload associated with RLT, resulting from the substantial volume of intravenous fluid administered during therapy (as assessed by changes in N-terminal pro-B-type Natriuretic Peptide (NT-proBNP levels)).

A detailed subgroup analysis was also performed, considering subpopulations potentially at higher risk for myocardial injury and exacerbation or new onset of cardiac dysfunction.

## 2. Materials and Methods

This retrospective observational study included all consecutive patients with progressive NETs (*n* = 60) who underwent RLT at the Department of Endocrinology and Radioisotope Therapy, Military Institute of Medicine—National Research Institute, Warsaw, Poland, between May 2023 and May 2025. Written informed consent for RLT was obtained from all patients in accordance with institutional protocols and ethical guidelines.

In accordance with institutional standards, prior to treatment, each patient underwent a comprehensive cardiologic assessment. This included a medical history focused on the diagnosis of CS, the presence of established CV diseases (with particular attention to HF and CHD), and current symptoms of HF (assessed according to the New York Heart Association (NYHA) classification). Additionally, CV risk factors were recorded, including age, hypertension, angina, diabetes mellitus, hyperlipidemia, smoking, obesity, and current medications. For each patient, the results of an echocardiographic examination performed within the preceding 6 months were assessed. In clinically indicated cases—such as the presence of symptoms suggestive of previously undiagnosed cardiac disease or worsening of symptoms since the last echocardiographic evaluation—a repeat echocardiogram was performed at the treating center prior to the initiation of RLT.

All patients underwent treatment with lutetium-based RLT, either as monotherapy (7400 MBq [^177^Lu]Lu-DOTA-TATE) (52 patients) or as tandem therapy with lutetium and yttrium [^177^Lu]Lu-DOTA-TATE/[^90^Y]Y-DOTA-TATE (administered in variable doses based on individual dosimetric data) (8 patients). Therapy was preceded by [^68^Ga]Ga-DOTA-TATE PET/CT or [^99m^Tc]Tc-HYNIC-TOC scintigraphy, in which high somatostatin receptor expression was confirmed in all tumor lesions in each patient. RLT courses were administered every 8 to 10 weeks. Between treatment courses, somatostatin analogs were administered every 4 weeks—either lanreotide at a dose of 120 mg or octreotide at a dose of 30 mg—with a mandatory 4-week discontinuation of long-acting somatostatin analogs prior to each RLT course.

During hospitalization, all patients received a standardized intravenous nephroprotective regimen consisting of amino acid (AA) infusions. The AA solution, at a concentration of 100 g/L, was administered in three doses: 500 mL immediately before the RLT procedure, another 500 mL concurrently during the RLT infusion via a separate venous access, and a final 500 mL dose on the day following radioisotope administration. Moreover, patients were given 1000 mL of a balanced electrolyte solution on the day of RLT and 500 mL of the same solution on the subsequent day. On the days when AAs were administered, patients also received intravenous antiemetic medication (ondansetron). In patients with pre-existing HF, the amount of intravenous fluids administered was not reduced; however, in the event of clinical symptoms, additional doses of loop diuretics were provided. Following each treatment course, post-therapeutic scintigraphy evaluation with SPECT/CT was performed.

### 2.1. Laboratory Tests

Blood samples were collected using BD Vacutainer^®^ tubes at the Department of Endocrinology and Radioisotope Therapy and processed at the Department of Medical Diagnostics, Military Institute of Medicine—National Research Institute, Warsaw, Poland.

All laboratory tests, including serum concentrations of high-sensitivity troponin I (troponin), CK-MB, and NTproBNP, were performed in the morning one day prior to RLT (troponin1, CK-MB1, NT-proBNP1) administration and again 48 h following the RLT procedure (troponin2, CK-MB2, NT-proBNP2). Based on the obtained results, the differences in troponin levels, CK-MB and NT-proBNP concentrations were calculated between samples collected 48 h post-treatment and one day prior to RLT administration (ΔTroponin, ΔNT-proBNP, ΔCK-MB, respectively).

Troponin and CK-MB concentrations were measured using electrochemiluminescence immunoassays (ECLIA) on the Cobas e411 analyzer (Roche Diagnostics, Mannheim, Germany). NT-proBNP concentrations were assessed using the Elecsys^®^ proBNP II assay (Roche Diagnostics, Mannheim, Germany), also run on the Cobas e411 platform. Serum creatinine concentrations were determined using enzymatic assays provided by Roche Diagnostics on the COBAS c503 PRO automated analyser (Hitachi High-Tech, Tokyo, Japan). Estimated glomerular filtration rate (eGFR) was calculated based on the CKD-EPI equation, incorporating age, sex, body weight, and serum creatinine concentration. Chromogranin A (CgA) concentrations were measured using enzyme-linked immunosorbent assays (ELISA) supplied by Labor Diagnostika Nord (LDN, Nordhorn, Germany), with a minimum detection limit of 1.4 µg/L. Complete blood counts were performed using a Sysmex XN-1000 automated hematology analyzer (Sysmex Corporation, Tokyo, Japan).

The reference ranges for the laboratory tests discussed in the paper are presented in Supplementary Table S1.

### 2.2. Echocardiography and Blood Pressure Measurement

Echocardiographic evaluations were performed using either the Vivid S70N or Vivid E95 systems (GE Healthcare, Chicago, IL, USA) by certified cardiologists in accordance with current echocardiography guidelines. Standard transthoracic echocardiography included quantitative and qualitative assessment of LVEF, left ventricular diastolic function (assessed by mitral inflow pattern, tissue Doppler imaging of mitral annulus velocity, and left atrial volume) right ventricular systolic function, including tricuspid annular plane systolic excursion (TAPSE), estimated right ventricular systolic pressure (RVSP), based on tricuspid regurgitant jet velocity and estimated right atrial pressure. All measurements were obtained and interpreted according to recommendations from the American Society of Echocardiography and the European Association of Cardiovascular Imaging.

Blood pressure was measured using a calibrated automated oscillometric sphygmomanometer (Omron M3 Comfort, Omron Healthcare, Kyoto, Japan), in a seated position after at least five minutes of rest, following ESC guidelines.

### 2.3. Statistical Analysis

Statistical analyses were conducted using IBM SPSS (version 23) and R software (version 4.3.1, R Core Team, 2023). The Shapiro–Wilk test was applied to assess the normality of data distribution. Variables demonstrating normal distribution were reported as means (M) with standard deviations (SD), whereas non-normally distributed data were expressed as medians (Med.) along with interquartile ranges (IQR). Group comparisons were performed using appropriate statistical tests, including Student’s *t*-test and the Mann–Whitney U test; prior to *t*-tests, Levene’s test was utilized to evaluate homogeneity of variances. A *p*-value less than 0.05 was considered statistically significant. Only complete patient datasets were included in the analyses. Additionally, univariable and multivariable linear regression analyses were performed to identify factors associated with biomarker changes, with multicollinearity assessed using the variance inflation factor (VIF).

## 3. Results

### 3.1. Patients’ Baseline Characteristics

**Figure 1:**
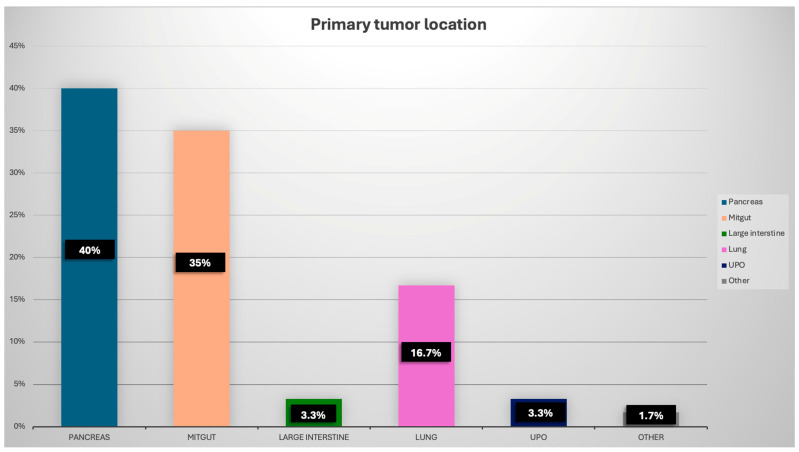
Primary localisation of NET. UPO—unknown primary origin.

A total of 60 patients (33 men and 27 women) with histologically confirmed metastatic, inoperable NET eligible for RLT were enrolled in the study. The median age was 66.5 years [IQR: 56.7–73.0]. The most common primary tumor locations were the pancreas (24 patients) and midgut (21 patients). Other sites included the lungs (10 patients), colon (2 patients), unknown primary origin (UPO) (2 patients), and stomach (1 patient). All patients in the study were receiving long-acting somatostatin analogs (octreotide or lanreotide). Nine patients had previously undergone RLT (median: 3 courses, range: 2–4). Sixteen patients had a history of CHT for NET, and four had been treated with sunitinib prior to the current therapy phase. Based on medical history, CS was diagnosed in 22 patients (36.7%), and CHD was documented in 4 patients (6.7%). One patient (1.7%) was found to have cardiac metastasis on pre-therapeutic [^68^Ga]Ga-DOTA-TATE PET/CT imaging. Chronic HF was diagnosed in 10 patients (16.7%): five were classified as NYHA class I and five as class II. The mean LVEF was 61% (range: 34–67%), while in the subgroup with HF, the mean LVEF was 48% (range: 34–55%). Other CV diseases included chronic coronary syndrome (CCS) (9 patients), atrial fibrillation (AF) (4 patients), chronic aortic aneurysm (2 patients), and a history of pulmonary embolism (PE) (1 patient). Baseline characteristics of the patients included in the study are presented in Table 1 and Figure 1.

Baseline echocardiographic data of the patients are presented in Supplementary Table S2.

The median baseline serum troponin1 concentration in the entire study population prior to the initiation of RLT was low and within the normal reference range, as was the median troponin1 concentration prior to all individual treatment courses combined. A slight elevation in the combined median troponin1 concentration prior to all RLT courses was observed in patients with CHD. The baseline median NT-proBNP1 level was elevated in patients with CHD, HF, and CCS, although a slight elevation from baseline values was also observed in the entire study group. Detailed values of serum troponin1, CK-MB and NT-proBNP1 concentrations are presented in Supplementary Table S3.

In the analysed cohort of 60 patients, the mean number of RLT courses administered was 3.6 (range: 2–4), with a total of 228 infusions performed. RLT was discontinued in 9 patients due to disease progression or other reasons unrelated to cardiological complications. In 4 patients with a history of prior RLT, the treatment plan initially included only 2 courses. A total of 52 patients received [^177^Lu]Lu-DOTA-TATE monotherapy, while 8 patients were treated with tandem therapy combining [^177^Lu]Lu-DOTA-TATE and [^90^Y]Y-DOTA-TATE. The mean cumulative activity of [^177^Lu]Lu-DOTA-TATE administered was 6899.0 ± 1462.6 MBq. Among those treated with [^177^Lu]Lu-DOTA-TATE monotherapy, the mean activity was 7402.9 ± 259.7MBq, whereas in the tandem therapy group, the mean activity of [^177^Lu]Lu-DOTA-TATE was 3019.5 ± 1020.8 MBq, and the mean activity of [^90^Y]Y-DOTA-TATE was 2263.8 ± 3885.6 MBq.

### 3.2. Clinical Tolerability of RLT

None of the patients reported new CV symptoms within the month preceding RLT initiation. All RLT administrations were well tolerated from a CV standpoint, with the exception of two cases: one patient experienced an ST-elevation myocardial infarction (STEMI) 24 h after RLT administration, and one female patient with pre-existing chronic HF, CS, and CHD developed a clinical exacerbation of HF symptoms. Both cases are described in detail below.

In none of the patients, apart from the two aforementioned cases, were any modifications made to chronic CV medications, including no increase in the dose or frequency of diuretic therapy.

The mean systolic blood pressure (SBP) prior to each RLT cycle was 133.0 mmHg (±8.0), while the mean diastolic blood pressure (DBP) was 80.0 mmHg (±7.0). Blood pressure measurements performed on the second day following RLT administration revealed a slight, non-significant decrease in mean SBP 131.0 mm Hg (±7.0); *p* = 0.36, along with a minor, clinically insignificant increase in mean DBP 82.0 mmHg (±6.0); *p* = 0.49.

### 3.3. Therapy-Related Renal, Hepatic, and Bone Marrow Toxicities

In the studied cohort, early statistically significant reductions in leukocyte, platelet, and neutrophil counts were observed in laboratory assessments performed 48 h after each course of RLT. Furthermore, when comparing complete blood count parameters obtained prior to the 1st and the 4th treatment course, a statistically significant decrease in leukocyte count—including both neutrophils and lymphocytes—as well as erythrocyte count, was also noted (Table 2 and Table S4).

Of note, no increase in median serum creatinine concentration or aspartate aminotransferase activity was observed in the study group, either in measurements performed before and 48 h after each RLT administration, or when comparing these parameters between the 1st and the 4th treatment cycles (Table 2 and Table S4).

### 3.4. Investigation of the Potential Cardiotoxicity Associated with RLT

**Figure 2:**
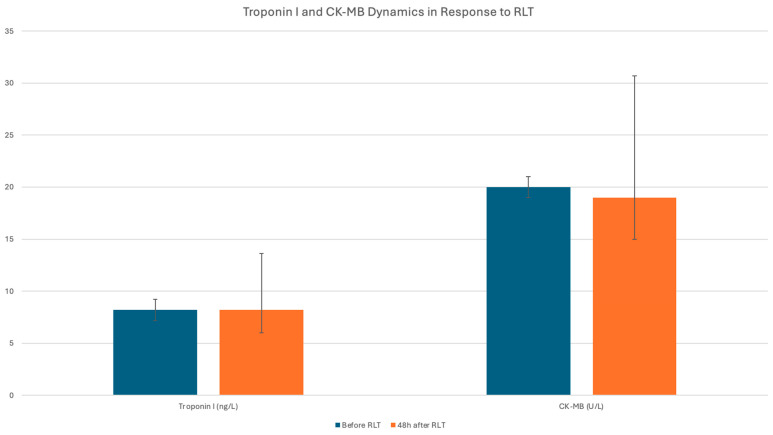
Changes in troponin I and CK-MB concentration in the entire study group across all RLT courses combined.

Based on the obtained results, no evidence of an adverse cardiotoxic effect of RLT on the myocardium was observed (Table 3 and Figure 2). Interestingly, in the entire study population, a small but statistically significant decrease in serum troponin concentration was noted 48 h after RLT administration. This effect was also present in the subgroup of patients treated with [^177^Lu]Lu-DOTA-TATE. Similarly, no cardiotoxic effects were observed in any of the evaluated subgroups, including patients with pre-existing HF, CHD, or those with a history of prior CHT or prior RLT. In most of the studied subgroups, a slight, clinically insignificant reduction in troponin concentration was observed, except in patients with CHD, where this pattern was not seen.

### 3.5. Effect of RLT on Heart Failure and Cardiac Overload

In the studied cohort, with the exception of a single case described below, RLT did not exacerbate HF symptoms nor lead to clinical or biochemical signs of cardiac overload. No worsening of functional status or progression in NYHA class was observed in patients with pre-existing HF. Similarly, no new-onset symptoms suggestive of volume overload, such as dyspnea, peripheral edema, or orthopnea, were reported.

**Figure 3:**
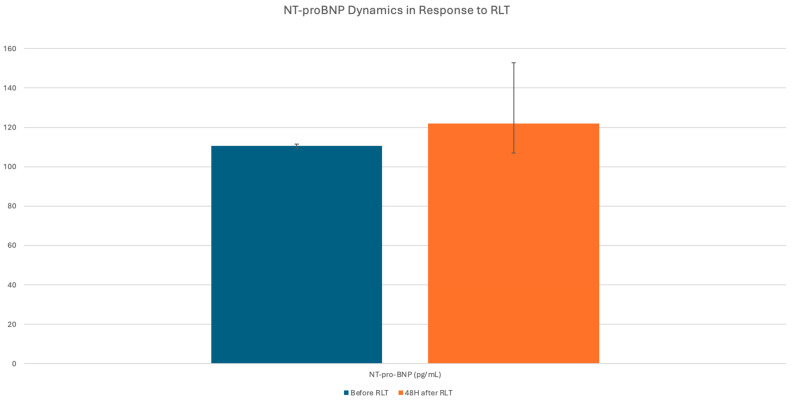
Changes in NT-proBNP concentration in the entire study group across all RLT cycles combined.

Biomarker analysis revealed no significant increase in NT-proBNP levels following RLT administration. In patients with CHD and those with HF as well as patients with prior CHT, NT-proBNP concentrations remained stable, with no clinically relevant deterioration noted during the treatment period (Table 4) (Figure 3).

When comparing troponin1, NT-proBNP1 and CK-MB1 concentration measured before the first and before the fourth course of RLT, no statistically significant differences in median values were observed. Similarly, no significant differences were found in the changes in troponin concentrations (ΔTroponin), CK-MB concentration (ΔCKMB), or NT-proBNP concentrations (ΔNT-proBNP) between the first and the final (fourth) treatment course (Supplementary Table S5).

### 3.6. Impact of Clinical and Biochemical Parameters on RLT-Associated Cardiotoxicity

No significant correlation was found between changes in troponin, CK-MB or NT-proBNP concentration and parameters such as patient age, BMI, baseline LVEF, TAPSE, or pre-treatment serum creatinine concentration (Supplementary Table S6).

Importantly, no correlation was also observed between median baseline NT-proBNP1 concentration (before each course) and subsequent changes in troponin concentration (ΔTroponin) or CK-MB concentration (ΔCK-MB) following RLT administration. Interestingly, a significant negative correlation was observed between baseline troponin1 concentrations (before each course) and the change in troponin concentration following each RLT administration (ΔTroponin) (r = −0.24, *p* = 0.004), as well as between baseline NT-proBNP1 concentration (before each cycle) and the corresponding change in NT-proBNP concentration after each treatment cycle (ΔNT-proBNP) (r = −0.41, *p* < 0.001). All correlations are presented in Supplementary Table S6.

Moreover, a statistically significant correlation was observed between the increase in serum troponin concentration (ΔTroponin) and the increase in serum creatinine concentration (Δcreatinine) following each RLT, suggesting a possible link between transient myocardial toxicity and renal impairment (r = 0.43, *p* < 0.001) (Table 5). No statistically significant correlation was found between the changes in leukocyte (ΔLeukocytes), erythrocyte (ΔErythrocytes), and platelet counts (ΔBlood platelets) and the changes in troponin (ΔTroponin) and CK-MB concentrations (ΔCK-MB) before and 48 h after each RLT course. A weak correlation was found between changes in NT-proBNP concentrations and lymphocyte as well as leukocyte counts. (Table 5).

No statistically significant correlation was observed between the administered activity of [^177^Lu]Lu-DOTA-TATE and changes in troponin, CK-MB or NT-proBNP concentration (*p* = 0.18, *p* = 0.31, and *p* = 0.06, respectively). Similarly, no significant correlations were found between the administered activity of [^90^Y]Y-DOTA-TATE and changes in these markers of cardiotoxicity (*p* = 0.88, *p* = 0.50, and *p* = 0.42, respectively).

In the univariable regression analysis, we examined the following potential predictors of troponin change: age, BMI, tumor localization, Ki-67 index, grading, prior chemotherapy, prior RLT, previous TKI treatment, CHD, HF, LVEF, β-blocker use, angiotensin-converting enzyme inhibitor (ACE-I) use, angiotensin II receptor blocker (ARB) use, baseline creatinine, as well as treatment type (monotherapy vs. tandem therapy). Among these, only Ki-67 (β = –0.213, *p* = 0.011), ARB use (β = –0.211, *p* = 0.012), ACE inhibitor use (β = 0.220, *p* = 0.008), and type of RLT (β = 0.139, *p* = 0.097) showed associations with troponin changes, whereas the other variables were not significant.

In the univariable analysis, several variables were tested as potential predictors of troponin change (ΔTroponin), including age, BMI, tumor localization, grading, Ki-67 index, type of RLT (monotherapy vs. tandem therapy), prior chemotherapy, prior RLT, prior TKI treatment, baseline LVEF, baseline creatinine concentration, β-blocker use, angiotensin-converting enzyme inhibitor (ACE-I) use, angiotensin II receptor blocker (ARB) use and comorbidities such as HF, CCS, hypertension, diabetes mellitus, and smoking status. Among these, the Ki-67 index (β = –0.213, *p* = 0.011), treatment with ACE-I (β = 0.220, *p* = 0.008), ARBs (β = –0.211, *p* = 0.012), and the presence of CHD (β = 0.168, *p* = 0.043) were statistically significant. In the multivariable analysis, Ki-67 (β = –0.212, *p* = 0.009), the use of tandem therapy (β = 0.260, *p* = 0.020), and treatment with ARBs (β = –0.284, *p* = 0.003) emerged as independent predictors of changes in troponin levels. In contrast, the presence of CHD did not remain statistically significant after adjustment for other covariates (β = 0.014, *p* = 0.889).

In the univariable regression analysis, the following variables were tested as potential predictors of changes in NT-proBNP concentration (ΔNT-proBNP): age, BMI, tumor location, Ki-67, tumor grading, RLT type (monotherapy vs. tandem therapy), prior chemotherapy, prior RLT, prior TKI, LVEF, use of beta-blockers, ACE-I, ARBs, diuretics, diabetes mellitus, arterial hypertension, smoking status, presence of CS, H, baseline creatinine, and baseline NT-proBNP concentrations. Among these, RLT type (tandem RLT) (β = 0.187, *p* = 0.026), beta-blocker use (β = –0.176, *p* = 0.037), and baseline NT-proBNP levels (β = –0.201, *p* = 0.016) showed significant associations with changes in NT-proBNP. In addition, the presence of CHD demonstrated a borderline association (β = 0.168, *p* = 0.043) and was considered clinically relevant. In the multivariable regression analysis, NT-proBNP concentration before RLT course (NT-proBNP1) was the only significant predictor of ΔNT-proBNP (β = –0.213, *p* = 0.022). The type of RLT demonstrated a borderline association (β = –0.209, *p* = 0.054), while the presence of CHD (β = 0.056, *p* = 0.60) and beta-blocker use (β = –0.093, *p* = 0.29) were not significantly associated with ΔNT-proBNP.

### 3.7. Clinically Overt Acute Cardiac Complications During Radioligand Therapy

During RLT, one patient with CCS and a history of a myocardial infarction 11 years prior to RLT—treated with coronary angioplasty—developed an acute inferior STEMI on the second day after the first RLT administration. Prior to treatment, the patient had no history of HF or CCS symptoms. The myocardial infarction was complicated by sudden cardiac arrest due to ventricular fibrillation. Importantly, in the period preceding the event, the patient had not been taking acetylsalicylic acid, beta-blockers, or statins chronically; despite cardiology recommendations and despite the ward’s standing prohibition, the patient smoked a cigarette immediately prior to the incident. Following successful resuscitation, an urgent coronary angiography revealed a critical stenosis in the right coronary artery. A successful percutaneous coronary intervention with implantation of everolimus-eluting stents in the right coronary artery was performed. Subsequent observation revealed no recurrence of chest pain. Follow-up transthoracic echocardiography demonstrated a decrease in LVEF to 43%, with symptoms of HF classified as NYHA class I. RLT was continued according to the original treatment plan, and the patient received three additional courses of RLT without further complications. No exacerbation of clinical signs of HF symptoms or significant increases in NT-proBNP were observed following the subsequent administrations. At the end of RLT, the echocardiographic assessment revealed a LVEF of 53%. In the over 20-year history of the department’s experience with RLT in patients with NET, this was the first cardiac event of this kind ever recorded.

The second patient in whom clinically overt cardiac complications were observed was a 32-year-old woman with an inoperable pancreatic NET G2, presenting with a large non-resectable abdominal mass, extensive hepatic and lymphatic metastases (involving both the thoracic and abdominal lymph nodes), CS, and cachexia (BMI 14.2, serum albumin 3.6 g/dL). The patient had a history of chronic biventricular HF (NYHA class II, LVEF 55%, TAPSE 18 mm) and CHD with significant tricuspid and pulmonary valve regurgitation. Following the first course of RLT, the patient experienced worsening dyspnea, ascites, marked peripheral edema, and an increase in NT-proBNP concentration (from 4327 to 6542 pg/mL), requiring the intensification of diuretic therapy. During the second treatment course, diuretics were administered from the first day of hospitalization, and only mild worsening of edema and dyspnea was observed. The patient discontinued RLT due to a progressive deterioration in her general condition and died within the following three months due to the progression of the disease.

### 3.8. Patient with NET Metastasis to the Heart

A 74-year-old female patient with a grade 2 neuroendocrine tumor (NET G2) of the small intestine and widespread metastases involving the liver, mesentery lymph nodes, skeleton, and myocardium was referred for RLT. Approximately 1.5 years prior to therapy, cardiac involvement was identified based on [^68^Ga]Ga-DOTA-TATE PET/CT, revealing metastatic lesions within the left ventricular wall. Echocardiography did not reveal the lesion; signs consistent with CHD were observed (moderate tricuspid insufficiency), with preserved global and segmental left ventricular systolic function.

**Figure 4:**
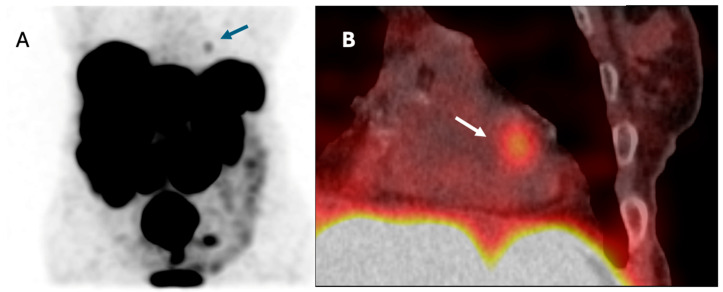
Post-therapeutic [^177^Lu]Lu-DOTA-TATE scintigraphy. (**A**)—[^177^Lu]Lu-DOTA-TATE planar scintigraphy, (**B**)—[^177^Lu]Lu-DOTA-TATE SPECT/CT. The cardiac metastasis is indicated by an arrow.

All treatment cycles were well tolerated, with no cardiovascular complaints reported. Importantly, post-therapy evaluations revealed no significant increases in serum concentrations of troponin, CK-MB, or NT-proBNP following any of the administered treatment courses (Supplementary Table S7). Post-therapeutic scintigraphy images are presented in Figure 4.

## 4. Discussion

NETs represent a heterogeneous group of neoplasms with variable clinical behavior, ranging from indolent to highly aggressive disease, and their accurate diagnosis is crucial for appropriate therapeutic decision-making. Imaging techniques, including contrast-enhanced CT, magnetic resonance imaging (MRI), and somatostatin receptor scintigraphy, play an important role in differentiating NETs from other pancreatic and gastrointestinal lesions [22,23,24]

RLT with [^177^Lu]Lu-DOTA-TATE and/or [^90^Y]Y-DOTA-TATE has been used for the treatment of NETs for over 30 years. Over time, growing evidence has confirmed both its safety and effectiveness in prolonging patient life [25,26,27,28]. Interestingly, despite widespread use, the CV safety of RLT has not yet been systematically assessed, unlike other oncological therapies, for which cardiotoxicity profiles are well-established. Both theoretical considerations and clinical data regarding the cardiotoxic effects of conventional chest radiotherapy suggest a potential for similar complications in RLT. However, most of the currently available data on RLT-related cardiotoxicity are limited to isolated case reports or small patient series. To date, only one published study has systematically evaluated the potential cardiotoxic effects of [^177^Lu]Lu-DOTA-TATE in NET patients. Although large prospective trials have not reported any deaths directly attributable to cardiac complications following RLT, rare case reports of cardiotoxicity do exist, highlighting the need for further investigation of this potential clinical concern [1,29,30,31].

The assessment of cardiac biomarkers, particularly troponin and NT-proBNP, continues to play a pivotal role in cardio-oncology. These biomarkers are capable of detecting subclinical myocardial injury even before the onset of clinical symptoms [32]. Numerous meta-analyses have confirmed the prognostic value of troponin in identifying patients at increased risk of left ventricular dysfunction, thereby reinforcing its clinical utility in the monitoring and early detection of cardiotoxicity in oncology patients. In a meta-analysis by Michel et al., which included 61 studies and a total of 5691 patients, elevated troponin levels were shown to be a strong predictor of left ventricular dysfunction, with an odds ratio of 11.9 [33]. Moreover, assessment of NT-proBNP has been shown to be a useful biomarker in cardio-oncology for early detection and monitoring of cardiac dysfunction during cancer treatment, as supported by recent guidelines and studies [4,34]. Additionally, elevated NT-proBNP levels have been correlated with subclinical cardiac injury and have demonstrated predictive value for subsequent development of cardiotoxicity in patients undergoing oncologic therapies [35,36].

The potential of ionizing radiation to adversely affect the CV system has been highlighted in a meta-analysis by Little et al., which examined the association between radiation exposure and CV diseases, as well as in a more recent comprehensive review by Jahng et al. [9,10]. Together, these studies emphasize the need for careful CV monitoring in patients undergoing radiotherapy due to the demonstrated increased risk of radiation-induced cardiac injury.

Data regarding both the short-term and long-term effects of radiotherapy delivered by ‘internal emitters’ remain scarce. Based on studies related to radioisotope therapy for thyroid cancer, Kolbert et al. demonstrated that the radiation dose absorbed by the blood may serve as a useful proxy for estimating the dose received by the CV system [11]. As demonstrated in the study by Little et al., a significant excess relative risk per Gray (Gy) was observed even for radiation exposures below 0.5 Gy [9]. In the case of radiopharmaceutical therapies, several studies have reported absorbed doses to the blood of at least 0.1 Gy per treatment course, with cumulative doses exceeding 0.3 Gy after multiple administrations [16,37,38,39]. These findings suggest that radiopharmaceutical therapies may adversely affect the CV system and increase the long-term risk of developing CV disease.

Dosimetric data regarding cardiac exposure during RLT for NETs also remain very limited. Based on the only available dosimetric analysis in this area, derived from the phase III NETTER-1 trial, the estimated absorbed dose to the cardiac wall during RLT with [^177^Lu]Lu-DOTA-TATE is approximately 0.03 mGy/MBq [15]. This value is similar to the absorbed dose to the red bone marrow, while the absorbed dose to the kidneys averages approximately 0.65 mGy/MBq. Given the similar incidence of nephrotoxic and myelotoxic complications observed with RLT, as with external beam radiotherapy, the toxic effects of RLT on tissues depend not only on the absorbed radiation dose but also on the intrinsic radiosensitivity of the affected tissue. Our findings support this concept, as despite the comparable estimated absorbed dose to the myocardium and bone marrow, clinically relevant myelotoxicity was observed, whereas no biochemical or clinical evidence of cardiotoxicity was detected. The relative radioresistance of the myocardium compared with bone marrow likely reflects fundamental biological differences: cardiomyocytes are terminally differentiated and comparatively resistant to DNA damage-induced apoptosis, whereas bone marrow harbors highly proliferative progenitor cells that are particularly sensitive to radiation exposure. Similarly, no correlation was observed between changes in hematologic parameters and changes in cardiotoxicity marker levels. The only correlation observed in the study was a weak positive correlation between NT-proBNP levels and leukocytes and lymphocyte counts, which may be related to systemic inflammatory responses or transient immune alterations induced by RLT, possibly occurring as part of the response to increased cardiac stress. This is an interesting observation that warrants further in-depth analysis.

Similarly to RLT in patients with NET, large studies evaluating the risk of early cardiotoxicity of radioiodine therapy in patients with differentiated thyroid cancer have not been published. The study by Stanciu et al. is one of the few that assess the impact of radioiodine therapy on cardiac function in thyroid cancer patients. Their results indicate an inverse correlation between the cumulative dose of ^131^I and LVEF (in patients without type 2 diabetes), suggesting a potential risk of cardiotoxicity associated with radioiodine therapy [40]. Growing evidence also suggests that radioiodine therapy may be linked to increased long-term CV risk, with outcomes potentially influenced by the cumulative administered dose [13].

So far, only one study has been published documenting early cardiotoxicity of RLT in patients with NET. In the study by Jafari et al., which included 13 NET patients undergoing 39 treatment courses, no significant increase in troponin I levels was observed [16]. Due to the small sample size, this study did not assess potential cardiotoxicity in subgroups of patients potentially at higher risk, such as those with diagnosed HF or CHD.

Since patients with NETs frequently receive other potentially cardiotoxic therapies a comprehensive evaluation of RLT’s CV safety profile may hold important clinical relevance. The CAPTEM regimen, commonly used in the treatment of NETs, appears to be generally well tolerated, with only sporadic reports of cardiac adverse effects even following prolonged treatment durations [41,42]. Nonetheless, capecitabine is associated with a 5–10% incidence of cardiotoxic events, ranging from silent ischemia and arrhythmias to, less frequently, serious complications such as myocardial infarction [43]. As such, cardiac monitoring—including electrocardiography and biomarker evaluation—is recommended during CAPTEM therapy, particularly in patients with preexisting CV diseases [44]. Also, sunitinib, a multi-TKI frequently used in the treatment of advanced NETs, has also been associated with a significant risk of cardiotoxicity, including hypertension, left ventricular dysfunction, and, in some cases, congestive HF [20,45].

In this retrospective analysis, 60 patients with diagnosed inoperable NETs underwent a total of 228 treatment courses (an average of 3.6 courses per patient). Consistent with the only previously published study on this topic, conducted by Jafari et al., no significant early cardiotoxicity related to RLT was observed, as assessed by serum troponin and NT-proBNP levels. There was no increase in troponin concentration following RLT administration, and in fact, a slight but statistically significant decrease in troponin concentration was observed in our study (Δtroponin −0.2 ng/L, *p* = 0.007). This recurrent observation of decreased troponin levels following treatment (as seen in both the study by Jafari et al. and ours) appears to have limited clinical relevance and is most likely attributable to the significant intravenous hydration administered during therapy. In our study, we additionally performed an analysis of cardiotoxicity in subgroups of patients at high risk of cardiotoxicity—an analysis that, to our knowledge, has not been conducted in any of the previously published studies, including the work by Jafari et al.

RLT with [^177^Lu]Lu-DOTA-TATE has become a well-established treatment option for patients with advanced NETs, owing to its favorable safety profile and efficacy. However, therapies involving [^90^Y]Y-DOTA-TATE, either alone or in tandem with [^177^Lu]Lu-DOTA-TATE, are often considered to be associated with a higher risk of organ toxicity. This is primarily attributed to the physical characteristics of yttrium-90, a high-energy β-emitter with a longer tissue penetration range (~11 mm vs. ~2 mm for lutetium-177), which may lead to increased off-target irradiation of healthy tissues, particularly the kidneys and bone marrow [46,47,48]. As such, tandem RLT ([^177^Lu]Lu-DOTA-TATE/[^90^Y]Y-DOTA-TATE), although explored as a strategy to enhance efficacy in patients with bulky or aggressive disease, has raised concerns regarding an elevated risk of treatment-related adverse effects, including hematologic and renal complications [49,50,51,52]. In our cohort, no clinical signs of cardiotoxicity were observed following tandem therapy, even in patients with pre-existing cardiovascular risk factors. However, it should be emphasized that tandem therapy emerged as an independent predictor of troponin changes in the multivariable analysis, which warrants caution and highlights the need for further evaluation of its potential cardiotoxic effects in larger patient cohorts.

Interestingly, in our study, no signs of cardiotoxicity were observed in key high-risk subpopulations, including patients with CS—where RLT administration may be associated with a sudden release of vasoactive substances—and those with CHD or pre-existing HF. Notably, individuals with established CV comorbidities, particularly those with HF, are generally recognized in cardio-oncology as being at significantly higher risk for treatment-related cardiotoxicity due to limited cardiac reserve and increased vulnerability to stressors such as CHT, targeted therapy, or radiotherapy [53,54,55]. Despite these theoretical risks, our cohort did not show significant elevations in cardiac biomarkers post-RLT, even in these fragile subgroups. To our knowledge, this is the first observation in the literature to report such findings in the context of RLT in NET patients. These observations suggest that RLT may have a more favorable cardiac safety profile than previously anticipated, even in vulnerable populations.

Interestingly, the Ki-67 index was inversely associated with troponin changes in the multivariable analysis (β = –0.212, *p* = 0.009). A potential explanation is that tumors with higher proliferation rates are typically characterized by lower uptake of radiolabeled somatostatin analogs, resulting in reduced retention of the radiopharmaceutical in high-avidity lesions. Consequently, the lower systemic exposure may translate into a reduced potential for myocardial injury and smaller increases in troponin levels.

Cardiac metastases from NETs are considered rare, with an estimated prevalence of approximately 2–4% based on modern imaging and autopsy series [56]. Although these lesions are often clinically silent, they may pose diagnostic and therapeutic challenges. To date, only isolated cases of patients with cardiac NET metastases experiencing complications after RLT have been described. Hendifar et al. reported a case of persistent cardiomyopathy following two administrations of [^177^Lu]Lu-DOTATATE in a patient with NET metastasis to the myocardium [29]. In our cohort, one patient was diagnosed with a metastatic NET to the left ventricle of the heart. Importantly, this patient underwent one full cycle (four courses) of tandem RLT ([^177^Lu]Lu-DOTA-TATE/[^90^Y]Y-DOTA-TATE) without experiencing clinical signs of cardiac dysfunction. Furthermore, there was no significant elevation in cardiac biomarkers following therapy. This observation is noteworthy, as it suggests that even in patients with direct cardiac involvement by NET, RLT may be safe from a cardiotoxicity standpoint.

In our cohort, we encountered a patient with established CCS who experienced an STEMI of the inferior wall 24 h after receiving the first course of RLT. To our knowledge, this is the first reported case of an acute coronary syndrome temporally associated with RLT. While previous literature has described cardiotoxic effects such as cardiomyopathy following RLT, no prior reports have documented an acute ischemic event of this nature [29]. Given the patient’s known atherosclerotic burden and smoking history, the infarction was likely precipitated by underlying coronary pathology rather than a direct effect of radionuclide exposure. Nonetheless, this case highlights the importance of comprehensive CV risk assessment and monitoring during RLT—particularly in older patients with pre-existing CV disease, who represent a growing proportion of the NET population.

NT-proBNP is a well-established and sensitive biomarker of myocardial wall stress, widely used in the evaluation of subclinical cardiac dysfunction in the context of oncological therapies. Current cardio-oncology guidelines issued by both the European Society of Cardiology (ESC) and the American Heart Association (AHA) recommend NT-proBNP assessment as part of routine surveillance for cardiotoxicity during cancer treatment, particularly in patients at increased risk of HF [4,54,55]. Elevated NT-proBNP concentrations may reflect either direct myocardial injury (and myocardial overload) or hemodynamic stress related to fluid overload. Although our present study did not reveal evidence of direct cardiotoxicity from RLT, it is important to acknowledge that RLT is associated with the administration of substantial volumes of intravenous fluids (over 3000 mL within 48 h), primarily due to the need for AA co-infusion and radiopharmaceutical dilution. Accordingly, guidelines advise caution in patients with pre-existing HF when undergoing RLT [4,57]. In our cohort, RLT was not associated with a statistically significant increase in NT-proBNP levels, either in the general population of patients with NETs or in high-risk subgroups, including those with known HF or CHD. To date, no studies have specifically evaluated changes in NT-proBNP following RLT in NET patients. Therefore, stable NT-proBNP concentrations indicate that RLT does not induce cardiotoxicity either through direct myocardial damage or through indirect fluid overload. Moreover, the current eligibility criteria for RLT—which exclude patients with advanced heart failure (NYHA class III–IV)—help ensure the selection of patients in whom the risk of treatment-related hemodynamic decompensation due to fluid infusion is minimized. Nevertheless, contrary to these findings, one patient in our study with biventricular HF and CHD did experience clinical deterioration following RLT, likely due to the relatively high-volume load in proportion to her low body mass and cachexia. This suggests that although RLT appears to be safe in terms of HF exacerbation in the overall population, particular caution is warranted in patients with significant pre-existing cardiac impairment.

This study has several limitations that should be acknowledged. First, it was conducted in a single-center setting with a relatively small sample size, which may limit the generalizability of our findings to broader NET populations. Moreover, this was a retrospective observational study. Conducting a prospective study with an additional control group (receiving only AA infusion and fluids in the same volume as the study group, without RLT) would allow for a more objective assessment of the results. Second, while the study assessed early changes in cardiac biomarkers within 48 h of RLT administration, it did not include long-term follow-up to evaluate delayed or cumulative cardiotoxic effects. Chronic or late-onset cardiotoxicity, which may be clinically relevant in NET patients receiving multiple treatment cycles, remains beyond the scope of this analysis.

Moreover, although echocardiographic evaluation was performed at baseline and clinically indicated during follow-up, advanced imaging modalities such as cardiac MRI or speckle-tracking echocardiography, which can detect subtle myocardial dysfunction, were not routinely employed. Another limitation is the lack of a control group not undergoing RLT, which would have provided a clearer comparator for assessing biomarker dynamics and clinical cardiac events.

Lastly, although efforts were made to analyse specific subgroups with increased CV risk—including patients with HF, CHD, or prior CHT or RLT exposure—the number of patients in these subgroups was small, limiting the statistical power to detect rare adverse events.

Despite these limitations, to the best of our knowledge, this is the first and largest study in the literature to comprehensively assess the potential cardiotoxicity of RLT in patients with NETs. Its findings are consistent with previously published smaller series and individual case reports, confirming the overall cardiac safety of this treatment. Importantly, this study is also the first to evaluate cardiac complications in predefined subgroups at increased CV risk, including patients with pre-existing HF, prior CHT, and previous radiotherapy. By systematically addressing this clinical issue and placing the results in the context of the available evidence, our work provides the most complete analysis to date and significantly extends current knowledge in the field.

An important future direction is the assessment of late and cumulative cardiotoxicity of RLT, particularly through the use of advanced imaging modalities such as cardiac MRI and speckle-tracking echocardiography. Given the inconclusive findings of the present study regarding the potential association between cardiotoxicity and the type of RLT, larger multicenter studies are warranted. Such investigations, including a greater number of patients treated with tandem RLT, will be essential to comprehensively evaluate the long-term cardiac safety of this therapeutic approach.

## 5. Conclusions

Our findings suggest that RLT, including tandem treatment with [^177^Lu]Lu-DOTA-TATE and [^90^Y]Y-DOTA-TATE, is a safe therapeutic option from a cardiotoxicity perspective in patients with NETs, even among patients with pre-existing CV comorbidities and increased susceptibility to such injury.

Furthermore, despite a comparable estimated absorbed dose delivered to the bone marrow and myocardium in dosimetric studies, no evidence of RLT-related cardiotoxicity was observed. This observation may reflect organ-specific differences in susceptibility to radiation damage, with the myocardium demonstrating greater resistance compared to hematopoietic tissue.

## Figures and Tables

**Table 1:** Baseline patients’ characteristics.

Parameter	Patients’ Characteristics
Age, years median (IQR)	66.5 (56.7–73.0)
Sex:	
Male, n (%)	33 (55.0)
Female, n (%)	27 (45.0)
Primary localisation of NET:	
Panceras, n (%)	24 (40)
Mitgut, n (%)	21 (35)
Large intestine, n (%)	2 (3.3)
Lung, n (%)	10 (16.7)
UPO, n (%)	2 (3.3)
Other, n (%)	1 (1.7)
Grade:	
G1, n (%)	18 (30.0)
G2, n (%)	40 (66.7)
G3, n (%)	2 (3.3)
Ki-67, median (IQR)	5 (2–10)
Prior RLT, n (%)	9 (15.0)
Prior CHT, n (%)	16 (26.7)
Prior treatment with TKI, n (%)	4 (6.7)
BMI, kg/m^2^, median (IQR)	25.1 (22.6–27.2)
Obesity (BMI ≥ 30), n (%)	8 (13.3)
Hypertension, n (%)	32 (53.3)
Diabetes mellitus, n (%)	19 (31.7)
Hyperlipidemia, n (%)	14 (23.3)
Smoking, n (%)	10 (16.7)
Carcinoid syndrome, n (%)	22 (36.7)
Carcinoid heart disease, n (%)	4 (6.7)
Cardiac metastases, n (%)	1 (1.7)
EF % (IQR)	61 (56–65)
Heart failure, n (%):	10 (16.7)
NYHA 1, n (%)	5 (8.3)
NYHA 2, n (%)	5 (8.3)
Other CVD, n (%):	14 (23.3)
Chronic coronary syndrome, n (%)	9 (15.0)
Atrial fibrillation, n (%)	4 (6.7)
Chronic aortic aneurysm, n (%)	2 (3.3)
History of pulmonary embolism, n (%)	1 (1.7)

BMI—body mass index; CHT—chemotherapy; CVD—cardiovascular diseases; IQR—interquartile ranges; NET—neuroendocrine tumor; NYHA—New York Heart Association; RLT—radioligand therapy; TKI—tyrosine kinase inhibitor; UPO—unknown primary origin.

**Table 2:** Comparison of hematologic parameters, serum creatinine concentration, and aspartate aminotransferase (AST) activity before and after each course of RLT.

Parameter	Before Each RLT Course	48 h after Each RLT Course	*p*
Leukocytes, ×10^9^/L	5.5 [4.3–6.7]	5.2 [4.0–6.8]	**0.049**
Erythrocytes, ×10^12^/L	4.0 [3.7–4.3]	4.0 [3.7–4.5]	0.18
Blood platelets, ×10^9^/L	198.0 [162.0–258.0]	185.0 [138.5–260.0]	**<0.001**
Neutrophils, ×10^3^/µL	3.4 [2.6–4.6]	3.2 [2.4–4.4]	**0.025**
Lymphocytes, ×10^3^/µL	1.1 [0.7–1.4]	1.0 [0.6–1.4]	0.11
Creatinine, mg/dL	0.9 [0.8–1.0]	0.9 [0.8–1.0]	0.37
GFR, mL/min/1.73 m^2^	79.3 [60.4–105.0]	79.9 [60.4—107.1]	0.56
AST, U/L	29.0 [24.0–39.0]	27.0 [21.0–35.0]	0.22

Data are presented as median and interquartile range. AST—aspartate aminotransferase; GFR—glomerular filtration rate; RLT—radioligand treatment.

**Table 3:** Effect of RLT on Troponin and CK-MB concentration.

	Number of Patients	Troponin1 (ng/L)	Troponin2 (ng/L)	ΔTroponin (ng/L)	*p*	CK-MB1 (U/L)	CK-MB2 (U/L)	ΔCK-MB (U/L)	*p*
All Pts	60	8.2 [5.8–13.6]	8.2 [6.0–13.6]	−0.2 [−1.4–0.3]	**0.007**	20.0 [16.0–32.7]	19.0 [15.0–30.7]	0.0 [−4.0–3.0]	0.90
Pts treated with [^177^Lu]Lu-DOTA-TATE	52	8.3 [5.7–13.4]	8.2 [5.9–13.7]	−0.1 [−1.3–0.3]	**0.008**	20.0 [16.0–32.7]	19.0 [15.2–30.7]	0.0 [−3.5–3.0]	0.57
Pts treated with tandem therapy	8	11.3 [6.1–16.6]	10.3 [6.3–15.3]	−0.7 [−1.7–1.3]	0.68	24.0 [15.2–51.0]	19.0 [14.0–45.7]	−0.5 [−10.7–3.0]	0.21
Pts with CS	22	8.3 [5.9–13.8]	8.6 [5.9–13.8]	−0.2 [−1.3–0.3]	0.10	18.0 [15.0–27.0]	18.0 [14.0–26.0]	0.0 [−0.4–3.0]	0.59
Pts with CHD	4	15.4 [5.8–19.1]	12.8 [5.9–22.6]	0.2 [−2.2–4.1]	0.68	58.0 [25.5–88.2]	43.5 [19.0–78.2]	−6.5 [−16.0–3.5]	0.33
All pts with HF	10	13.5 [11.6–20.7]	13.3 [10.4–21.7]	−0.7 [−2.5–0.8]	0.40	28.5 [17.7–45.0]	30.5 [17.7–45.7]	0.0 [−6.7–13.5]	0.62
HF NYHA I	5	11.8 [10.7–18.9]	12.8 [7.9–18.8]	−0.7 [−3.0–0.65]	0.21	21.0 [14.0–20.0]	19.0 [17.0–43.0]	1.0 [−4.0–19.5]	0.67
HF NYHA II	5	16.7 [12.9–21.1]	16.2 [10.8–25.6]	−0.8 [−1.6–1.8]	0.65	37.0 [21.7–56.0]	33.5 [21.5–59.7]	−0.5 [−9.7–4.7]	0.89
Pts with CCS	9	13.8 [11.0–22.5]	13.7 [9.9–24.0]	−0.45 [−1.5–0.8]	0.45	26.5 [17.0–37.2]	26.5 [15.7–49.2]	−0.5 [−7.2–3.5]	0.90
Pts with prior RLT	9	9.0 [7.4–16.0]	9.8 [6.1–15.6]	−0.8 [−1.7–0.3]	0.12	28.0 [21.0–75.5]	22.0 [17.0–77.0]	−1.0 [−8.0–34.0]	0.88
Pts with prior CHT	16	5.7 [3.6–7.2]	4.5 [3.1–8.1]	−0.1 [−0.6–0.15]	0.40	12.0 [11.0–16.0]	15.0 [10.0–28.0]	1.0 [0.0–5.2]	0.08
Pts with prior TKI	4	7.1 [4.9–9.8]	6.7 [4.9–7.8]	−0.5 [−3.0–0.3]	0.11	21.0 [18.0–34.5]	19.5 [18.7–29.2]	0.5 [−4.5–6.7]	0.68

Data are presented as median and interquartile range. *p*—statistical significance between measurement “1” (prior to RLT administration) and measurement “2” (48 h post-RLT administration). CHD—carcinoid heart disease; CHT—chemotherapy; CCS—chronic coronary syndrome; CS—carcinoid syndrome; HF—heart failure; NYHA—New York Heart Association; Pts—patients; RLT—radioligand therapy; TKI—tyrosine kinase inhibitor.

**Table 4:** Effect of RLT on NT-proBNP concentration.

	Number of Patients	NT-proBNP1 (pg/mL)	NT-proBNP2 (pg/mL)	ΔNT-proBNP (pg/mL)	*p*
All Pts	60	117.5 [40.5–319.7]	121.5 [37.9–322.2]	−4.0 [−45.6–33.6]	0.32
Pts treated with [^177^Lu]Lu-DOTA-TATE	52	106.0 [28.1–316.0]	127.0 [33.2–350.5]	−4.0 [−46.0–33.6]	0.53
Pts treated with tandem therapy	8	208.0 [103.0–358.5]	185.0 [69.7–295.0]	−21.6 [−44.1–16.7]	0.08
Pts with CS	22	191.1 [58.2–410.0]	187.0 [55.3–359.0]	−0.5 [−46.0–34.7]	0.49
Pts with CHD	4	430.0 [224.0–3644.0]	301.0 [256.0–1925.0]	−7.0 [−290.0–130.0]	0.79
All pts with HF	10	531.5 [273.2–1166.7]	659.0 [294.0–1255.0]	−14.8 [−145.7–142.5]	0.64
Pts with HF NYHA I	5	624.0 [166.0–1039.5]	615.0 [107.2–906.5]	−92.0 [−317.5–111.7]	0.26
Pts with HF NYHA II	5	531.5 [296.0–2026.0]	870.0 [297.0–1333.0]	18.5 [−83.0–260.0]	0.51
Pts with CCS	9	306.5 [115.5–609.0]	274.0 [208.0–569.0]	0.0 [−214.5–129.5]	0.76
Pts with prior RLT	9	207.5 [15.8–303.7]	283.0 [211.0–326.0]	−4.4 [−37.3–116.7]	0.33
Pts with prior CHT	16	47.4 [17.8–63.3]	64.4 [30.0–118.0]	9.9 [−3.6–52.7]	0.18
Pts with prior Other CVD	4	79.9 [44.3–120.5]	60.7 [31.7–316.7]	−17.4 [−30.5–216.4]	0.65

Data are presented as median and interquartile range. *p*—statistical significance between measurement “1” (prior to RLT administration) and measurement “2” (48 h post-RLT administration). CHD—carcinoid heart disease; CHT—chemotherapy; CCS—chronic coronary syndrome; CS—carcinoid syndrome; HF—heart failure; NYHA—New York Heart Association; Pts—patients; RLT—radioligand therapy.

**Table 5:** Correlations between markers of cardiotoxicity.

	ΔTroponin	ΔCK-MB	ΔNT-proBNP
Δcreatinine	r = 0.43 *p* < 0.001	*p* = 0.90	*p* = 0.83
ΔGFR	*p* = 0.95	*p* = 0.63	*p* = 0.73
ΔTroponin	-	*p* = 0.95	*p* = 0.53
ΔCK-MB	*p* = 0.95	-	*p* = 0.23
ΔLeukocytes	*p* = 0.92	*p*= 0.89	r = 0.39 *p* <0.001
ΔErythrocytes	*p* = 0.67	*p* = 0.30	*p*= 0.96
ΔBlood platelets	*p* = 0.47	*p* = 0.88	*p* = 0.59
ΔNeutrophils	*p* = 0.64	*p* = 0.93	*p* = 0.87
ΔLymphocytes	*p* = 0.44	*p* = 0.34	r = 0.20 *p* = 0.047
ΔAST	*p* = 0.59	r = 0.31 *p* < 0.001	*p* = 0.11

ΔAST—change in serum AST activity between the measurement taken prior to RLT administration and 48 h post-treatment; ΔBlood platelets—change in blood platelets count between the measurement taken prior to RLT administration and 48 h post-treatment; Δcreatinine—change in serum creatinine concentration between the measurement taken prior to RLT administration and 48 h post-treatment; ΔGFR—change in GFR between the measurement taken prior to RLT administration and 48 h post-treatment; ΔCK-MB—change in serum CK-MB activity between the measurement taken prior to RLT administration and 48 h post-treatment; ΔLymphocytes—change in lymphocytes count between the measurement taken prior to RLT administration and 48 h post-treatment; ΔNeutrophils—change in neutrophils count between the measurement taken prior to RLT administration and 48 h post-treatment; Δcreatinine ΔNT-proBNP—change in serum NT-proBNP concentration between the measurement taken prior to RLT administration and 48 h post-treatment; ΔTroponin—change in serum troponin concentration between the measurement taken prior to RLT administration and 48 h post-treatment. *p*—statistical significance; r—correlation coefficient.

## Data Availability

The datasets used and/or analyzed during the current study are available from the corresponding author on reasonable request.

## Supplementary Materials

The following supporting information can be downloaded at: https://www.mdpi.com/article/10.3390/cancers17193219/s1, Table S1: Reference Ranges for Biochemical and Hematological Parameters. Table S2: Echocardiographic parameters of patients qualified for the study. Table S3: Baseline serum concentrations of markers of cardiotoxicity. Table S4: Comparison of hematologic parameters, serum creatinine levels, and aspartate aminotransferase (AST) activity before the first and fourth RLT courses. Table S5: Measurements of markers of cardiotoxicity and changes in their levels before the first and before the fourth course of RLT. Table S6: Impact of clinical and biochemical parameters on changes in levels of markers of cardiotoxicity assessed before and 48 hours after each course of RLT. Table S7: Markers of cardiotoxicity in the patient who experienced heart failure exacerbation during RLT.

## Abbreviations

The following abbreviations are used in this manuscript:

AA	Amino acid
ACE-I	Angiotensin-converting enzyme inhibitor
AF	Atrial fibrillation
AHA	American Heart Association
ARB	Angiotensin II receptor blocker
AST	Aspartate aminotransferase
BMI	Body mass index
BP	Blood pressure
CAPTEM	Capecitabine/temozolomide
CCS	Chronic coronary syndrome
CHD	Carcinoid heart disease
CHT	Chemotherapy
CgA	Chromogranin A
CK-MB	Creatine Kinase—MB isoenzyme
CS	Carcinoid syndrome
CV	Cardiovascular
CVD	Cardiovascular disease
DBP	Diastolic blood pressure
ELISA	Enzyme-linked immunosorbent assay
ESC	European Society of Cardiology
GFR	Glomerular filtration rate
GLS	Global longitudinal strain
HF	Heart failure
ICIs	Immune checkpoint inhibitors
IC-OS	International Cardio-Oncology Society
IQR	Interquartile range
LVEF	Left ventricle ejection fraction
M	Mean
Med	Median
MRI	Magnetic resonance imaging
mTOR	Mammalian Target of Rapamycin Inhibitors
NET	Neuroendocrine tumor
NT-proBNP	N-terminal pro-B-type Natriuretic Peptide
NYHA	New York Heart Association
PE	Pulmonary embolism
RLT	Radioligand therapy
RVSP	Right ventricular systolic pressure
SBP	Systolic blood pressure
SD	Standard deviations
STEMI	ST-elevation myocardial infarction
TAPSE	Tricuspid annular plane systolic excursion
TKI	Tyrosine kinase inhibitors
Troponin	Troponin I
UPO	Unknown primary origin
VIF	Variance inflation factor
