# Supplementary material for: Assessment of Early Cardiotoxicity and Cardiac Dysfunction of Radioligand Therapy in Patients with Neuroendocrine Tumors
Source: Cancers (Basel). 2025 Oct 2;17(19):3219. doi: 10.3390/cancers17193219 (PMC12524008; doi:10.3390/cancers17193219)
Supplement: Supplementary file 1 [file cancers-17-03219-s001.zip › cancers-3888819-supplementary.pdf]

cancers

MDPI

Article

Assessment of Early Cardiotoxicity and Cardiac Dysfunction of Radioligand Therapy in Patients with Neuroendocrine Tumors

Katarzyna Jóźwik-Plebanek [1,*], Marek Saracyn [1,2], Maciej Kołodziej [1], Weronika Mądra [1], Adam Daniel Durma [1,2], Mirosław Dziuk [3], Zuzanna Balcerska [1], Katarzyna Janiak [1], Katarzyna Gniadek-Olejniczak [4] and Grzegorz Kamiński [1]

1 Department of Endocrinology and Radioisotope Therapy, Military Institute of Medicine—National Research Institute, 04-141 Warsaw, Poland; msaracyn@wim.mil.pl (M.S.); mkolodziej@wim.mil.pl (M.K.); wmadra@wim.mil.pl (W.M.); adurma@wim.mil.pl (A.D.D.); zbalcerska@wim.mil.pl (Z.B.); kjaniak@wim.mil.pl (K.J.); gkaminski@wim.mil.pl (G.K.)
2 Faculty of Medicine, Warsaw University, 00-927 Warsaw, Poland
3 Nuclear Medicine Department, Military Institute of Medicine—National Research Institute, 04-141 Warsaw, Poland; mdziuk@wim.mil.pl
4 Neurorehabilitation Clinic, Military Institute of Medicine—National Research Institute, 04-141 Warsaw, Poland; kgniadek-olejniczak@wim.mil.pl
* Correspondence: kjozwik-plebanek@wim.mil.pl; Tel.: +48-261816110

Supplementary Table S1. Reference Ranges for Biochemical and Hematological Parameters.

Parameter	Unit	Reference Range
Troponin	ng/L	0.0–14.0
CK-MB	U/L	0.0 – 25.0
NT-pro-BNP	pg/mL	0.0 - 125.0
Creatinine	mg/dL	0.7 - 1.2
GFR	mL/min/1.73 m²	>60
AST	U/L	>36
Leukocytes	×10^9/L	4.30 - 9.64
Erythrocytes	×1012/L	4.36 - 5.78
Blood platelets	×10^9/L	163 - 347
Neutrophils	×10^3/μL	1.93 - 5.87
Lymphocytes	×10^3/μL	1.23 - 3.42
Chromogranin A	ng/mL	<100

AST—aspartate aminotransferase; CK-MB — Creatine Kinase—MB isoenzyme; GFR—glomerular filtration rate.

Supplementary Table S2. Echocardiographic parameters of patients qualified for the study.

Parameter	Data	Unit	Reference Range
LVEDD, median (IQR)	51.7 (46.7–55.9)	mm	39–52
LVESD, median (IQR)	34.8 (28.9–39.5)	mm	25–40
EF % (IQR)	61.0 (56.0–65.0)	%	55–70
TAPSE, median (IQR)	21.0 (19.0–22.0)	mm	>16
IT, n (%)	7 (11.7)	-	-
IP, n (%)	3 (5.0)	-	-

EF—ejection fraction; IQR—interquartile range; IP—pulmonary valve regurgitation (moderate or severe); IT—tricuspid valve regurgitation (moderate or severe); LVEDD—left ventricle end diastolic diameter; LVESD—left ventricle end systolic diameter; TAPSE—tricuspid annular plane systolic excursion.

Supplementary Table S3. Baseline serum concentrations of markers of cardiotoxicity.

	Troponin1 ng/L	CK-MB1 U/L	NT-proBNP 1 pg/mL
Before start of RLT	10.0 [5.8–16.0]	25.5 [17.2–35.7]	158.0 [54.1–365.0]
Before each RLT administration	8.2 [5.8–13.6]	20.0 [16.0–32.7]	110.5 [33.6–300.5]
Pts with CS, before each RLT administration	8.3 [5.9–13.8]	18.0 [15.0–27.0]	191.0 [58.2–410.0]
Pts with CHD, before each RLT administration	15.4 [5.8–19.1]	58.0 [25.5–88.2]	430 [224.0–3644.0]
Pts with HF, before each RLT administration	13.1 [11.3–20.8]	33.0 [19.0–45.0]	580.0 [257.7–1228.2]
Pts with CCS, before each RLT administration	13.0 [11.3–26.6]	27.0 [18.0–45.0]	214.0 [90.-672.0]

Data are presented as median and interquartile range. CHD—carcinoid heart disease; CCS—chronic coronary syndrome; CK-MB — Creatine Kinase—MB isoenzyme; CS—carcinoid syndrome; HF—heart failure; IQRs—interquartile ranges; Pts—patients; RLT—radioligand treatment.

Supplementary Table S4. Comparison of hematologic parameters, serum creatinine levels, and aspartate aminotransferase (AST) activity before the first and fourth RLT courses.

	Before first RLT Course	Before 4th RLT Course	*p*
Leukocytes, ×10^9/L	6.4 [5.3–7.5]	4.5 [3.9–6.4]	0.004
Erythrocytes, ×1012/L	4.3 [3.9–4.6]	3.9 [3.5–4.1]	0.002
Blood platelets, ×10^9/L	223.5 [153.7–293.0]	182.0 [146.5–254.0]	0.190
Neutrophils, ×10^3/µL	4.0 [3.0–5.1]	3.0 [2.1–4.3]	0.039
Lymphocytes, ×10^3/µL	1.4 [1.1–2.0]	0.8 [0.6–1.3]	<0,001
Creatinine, mg/dL	0.9 [0.8–1.0]	0.9 [0.8–1.1]	0.670
GFR, mL/min/1.73m2	84.8 [58.4–109.7]	73.7 [57.8–100.8]	0.780
AST, U/L	29.5 [20.0–48.0]	30.0 [26.8–37.0]	0.260

Data are presented as median and interquartile range. AST—aspartate aminotransferase; GFR—glomerular filtration rate; RLT—radioligand treatment.

Supplementary Table S5. Measurements of markers of cardiotoxicity and changes in their levels before the first and before the fourth course of RLT.

	First RLT Course	4th RLT Course	*p*
Troponin, ng/L	10.0 [5.8–16.0]	7.9 [6.2–12.1]	0.140
CK-MB, U/L	25.5 [17.2–35.7]	7.9 [6.2–12.1]	0.070
NT-pro-BNP, pg/mL	158.0 [54.1–365.0]	106.0 [35.0–316.0]	0.780
ΔTroponin, ng/L	-0.7 [-1.8–0.2]	-0.1 [-1.7–0.8]	0.810
CK-MB, U/L	0.0 [-4.7–6.7]	0.0 [-3.0–2.5]	0.600
ΔNT-pro-BNP, pg/mL	-2.6 [-92.0–28.2]	-27.6 [-66.8- -0.7]	0.850

Data are presented as median and interquartile range. ΔCK-MB—change in serum CKMB activity between the measurement taken prior to RLT administration and 48 hours post-treatment; ΔNT-proBNP—change in serum NT-pro-BNP concentration between

the measurement taken prior to RLT administration and 48 hours post-treatment; ΔTroponin—change in serum troponin concentration between the measurement taken prior to RLT administration and 48 hours post-treatment; P—statistical significance.

Supplementary Table S6. Impact of clinical and biochemical parameters on changes in levels of markers of cardiotoxicity assessed before and 48 hours after each course of RLT.

	**ΔTroponin**	**ΔCK-MB**	**ΔNT-proBNP**
Age	r = 0.13<br>p = 0.13	r = 0.02<br>p = 0.82	r = -0.10<br>p = 0.230
BMI	r = -0.8<br>p = 0.37	r = 0.10<br>p = 0.22	r = 0.06<br>p = 0.48
Troponin1 concentration before each RLT course	r = -0.24<br>p = 0.004	r = 0.02<br>p = 0.79	r = -0.10<br>p = 0.25
CK-MB1 level before each RLT course	r = 0.02<br>p = 0.79	r = 0.51<br>p < 0.005	r = -0.04<br>p = 0.67
NT-pro-BNP1 concentration before each RLT course	r = 0.05<br>p = 0.57	r = 0.12<br>p = 0.17	r = -0.41<br>p < 0.001
Creatinine concentration before each RLT course	r = 0.005<br>p = 0.96	r = -0.09<br>p = 0.29	r = -0.32<br>p = 0.70
GFR before each RLT course	r = -0.13<br>p = 0.13	r = 0.10<br>p = 0.26	r = 0.12<br>p = 0.14
AST level before each RLT course	r = -0.10<br>p = 0.90	r = 0.51<br>p < 0.001	r = 0.04<br>p = 0.61
Chromogranin A concentration before each RLT course	r = -0.08<br>p = 0.33	r = 0.39<br>p < 0.001	R = 0.03<br>p = 0.76
LVEF before RLT	r = -0.13<br>p = 0.13	r = 0.004<br>p = 0.96	r = 0.09<br>p = 0.30
TAPSE before RLT	r = -0.06<br>p = 0.44	r = 0.06<br>p = 0.45	r = 0.07<br>p = 0.44

ΔCKMB—change in serum CK-MB activity between the measurement taken prior to RLT administration and 48 hours post-treatment; ΔNT-pro-BNP—change in serum NT-proBNP concentration between the measurement taken prior to RLT administration and 48 hours post-treatment; ΔTroponin—change in serum troponin concentration between the measurement taken prior to RLT administration and 48 hours post-treatment; p—statistical significance; **r**—correlation coefficient. AST—aspartate aminotransferase**;** BMI—body mass index; LVEF—left ventricular ejection fraction; RLT—radioligand treatment; TAPSE—tricuspid annular plane systolic excursion.

Supplementary Table S7. Markers of cardiotoxicity in the patient who experienced heart failure exacerbation during RLT.

	Troponin1 (ng/L)	Troponin2 (ng/L)	CK-MB1 (U/L)	CK-MB2 (U/L)	NT-proBNP1 (pg/mL)	NT-proBNP2 (pg/mL)
I cycle of RLT 3500/1885 MBq [^{177}Lu]Lu/[^{90}Y]Y-DOTA-TATE	18.7	26.1	29.0	19.0	364.0	293.0
II cycle of RLT 3500/400 MBq [^{177}Lu]Lu/[^{90}Y]Y-DOTA-TATE	17.0	20.1	38.0	19.0	273.0	301.0
III cycle of RLT 3500/530 MBq [^{177}Lu]Lu/[^{90}Y]Y-DOTA-TATE	17.2	31.8	21.0	26.0	224.0	354.0
IV cycle of RLT 3500/600 MBq [^{177}Lu]Lu/[^{90}Y]Y-DOTA-TATE	20.3	21.5	27.0	30.0	430.0	395.0

RLT—radioligand treatment.
